# Evaluation of a male-specific psychotherapeutic program for major depressive disorder compared to cognitive behavioral therapy and waitlist: study protocol for a six-arm randomized clinical superiority trial examining depressed eugonadal and hypogonadal men receiving testosterone

**DOI:** 10.3389/fpsyt.2023.1129386

**Published:** 2023-06-21

**Authors:** Andreas Walther, Ulrike Ehlert, Michèle Schneeberger, Lukas Eggenberger, Christoph Flückiger, Nikola Komlenac, Adrian Heald, Timothy Rice, Simona Palm, Zac E. Seidler, John S. Ogrodniczuk, John L. Oliffe, Simon M. Rice, David Kealy, Rainer Weber, David Zimmermann

**Affiliations:** ^1^Department of Clinical Psychology and Psychotherapy, University of Zurich, Zurich, Switzerland; ^2^Department of Psychology, University of Kassel, Kassel, Germany; ^3^Institute of Diversity in Medicine, Medical University of Innsbruck, Innsbruck, Austria; ^4^Department of Endocrinology, University of Manchester, Manchester, United Kingdom; ^5^Department of Psychiatry, Icahn School of Medicine at Mount Sinai, New York, NY, United States; ^6^Orygen, Parkville, VIC, Australia; ^7^Centre for Youth Mental Health, The University of Melbourne, Melbourne, VIC, Australia; ^8^Department of Psychiatry, University of British Columbia, Vancouver, BC, Canada; ^9^School of Nursing, University of British Columbia, Vancouver, BC, Canada; ^10^Department of Nursing, The University of Melbourne, Melbourne, VIC, Australia; ^11^Faculty of Medicine and University Hospital Cologne, Clinic and Polyclinic for Psychosomatics and Psychotherapy, University of Cologne, Cologne, Germany; ^12^Andrology and Urology Centre, Uroviva Network, Zurich, Switzerland

**Keywords:** major depressive disorder, psychotherapy, hypogonadism, testosterone treatment, traditional masculinity ideologies, psychotherapy effectiveness, psychotherapy utilization, men’s mental health

## Abstract

**Background:**

Treatment of major depressive disorder (MDD) in men is complicated by the endorsement of traditional masculinity ideologies (TMI) often leading to reluctance toward psychotherapy, therapy interfering processes, or premature termination. In addition, it has been shown that men with MDD have a significantly increased risk of being hypogonadal (e.g., total testosterone levels <12.1 nmoL/L). Therefore, it is recommended to examine depressed men with regard to their testosterone status and if hypogonadism is present to combine psychotherapy with testosterone treatment (TT).

**Aim:**

This project aims to evaluate a male-specific psychotherapeutic program (MSPP) for MDD in depressed eugonadal and hypogonadal men receiving testosterone in comparison to a standard cognitive behavioral therapy (CBT) for MDD and a Waitlist.

**Methods:**

The study presents a 2×3 factorial study design. In total, 144 men aged between 25 and 50 will be stratified by testosterone status (eugonadal/hypogonadal) and then randomized into one of the three conditions (MSPP, CBT, or Waitlist). Additionally, a healthy control group of 100 men will be recruited, which will undergo only baseline assessments. Both standardized psychotherapy programs will encompass 18 sessions delivered in a weekly manner. Aligned with the TT-related medical visits of the 72 hypogonadal men, all participants will be followed up with clinical assessments and bio sampling at weeks 0, 6, 15, 24, and 36.

**Expected results:**

Compared to Waitlist control groups, treatment groups are expected to be more effective and efficacious (depression score reduction of ≥50%) at week 24 and at the follow-up at week 36. The MSPP is expected to show higher effectiveness and efficacy for depressive symptoms and higher acceptability (lower dropout rate) as compared to CBT.

**Discussion:**

This study represents the first attempt to test a male-specific psychotherapy for MDD in a single-setting compared to standard CBT and a Waitlist control condition using randomized clinical trial methodology. In addition, the potential positive adjunct effect of psychotherapy to TT in reducing depressive burden and improving quality of life in hypogonadal depressed men represents a neglected research area and might introduce new hypogonadism screening procedures in depressed men and combined treatment approaches for depressed men suffering from hypogonadism. Limitations are the rigorous inclusion and exclusion criteria, which limit the generalizability of the study results to first episode treatment naïve depressed men.

**Clinical Trial Registration:**

ClinicalTrials.gov, identifier NCT05435222.

## 1. Introduction

Major depressive disorder (MDD) is the leading cause of disability worldwide (1). There are major difficulties in reducing the high prevalence rates of depression and improving its treatment (2, 3). MDD shows high 12-month and lifetime prevalence for women of 13.4 and 26.1% and for men of 7.2 and 14.7% (4). The marked gender differences in prevalence rates led researchers to formulate biologically informed models discussing reduced depression vulnerability in men (5–10). Yet, a rapidly growing body of research suggests that the much lower observed depression prevalence rates in men is related to men’s endorsement of traditional masculinity ideologies (TMI) (11–14).

TMI are socially defined sets of standards and norms how men are expected to be or behave (15, 16). High endorsement of TMI such as restrictive emotionality, self-reliance, or dominance make it difficult for men to communicate depressive feelings, seek help, and engage in psychotherapy (17–21). Multiple studies report that many men with depression exhibit a characteristic alternative symptom profile marked by externalizing symptomatology such as anger, irritability, risk taking, or substance abuse (22–25). Men further show over 30% lower psychotherapy use when suffering from depression as compared to women (18, 20, 26–29). High endorsement of TMI has consistently been related to worse mental health outcomes and reduced psychotherapy uptake (21). Strong endorsement of TMI is further linked to increased self-stigmatization in depressed men and to the feeling that a man should be able to cope with mental health problems without professional help (30). It is therefore not surprising that in a longitudinal observational study over 20 years, men with high endorsement of TMI died twice as much by suicide as men exhibiting low endorsement of TMI (31) This is alarming since men in general die by suicide up to four times more often than women (32). Furthermore, men with high endorsement of TMI are particularly vulnerable to commit suicide in v response to stressors such as economic crises (33). Notably, over 60% of men, who died by suicide show a contact with a mental health specialist in the 12 months prior suicide (34). Since depression is one of the prime risk factors for suicide, these studies highlight that many men suffering from depression do try to find help for their mental health issues, but often do not or only insufficiently engage in treatment.

Investigating the roots of these gender differences, researchers identified gender role socialization as the starting point of these gender-aligned psychopathology. Being born male or female is one of the most defining distinctions in our society, and gender-appropriate behavior is promoted in children at an early age and gender-non-compliant behavior is commonly sanctioned (35). Furthermore, it is relevant that although girls and boys are encouraged to behave in a gender-conforming manner (36), boys are punished more severely for gender-inappropriate behavior (35, 37). This pattern persists into adulthood (38). As mentioned above, TMI are a socially constructed and idealized set of expectations and behaviors embodied in the individual man and culturally regarded as appropriate to males (39–41). Based on their gender role identity as male, boys and men are socially reinforced to conform to TMI with the two main foci “be in control” and “be unlike women” (15, 16).

Suffering from a depression, which is commonly associated with losing control, emotionality and femininity, therefore stands opposed to TMI. Studies highlight that suffering from depressive disorders is often associated with the experience of public and self-stigma, especially when men endorse TMI (42, 43). It was further shown that men suffering from depression are perceived by healthy others as less masculine and more feminine (44) While depression is perceived as feminine, psychotherapy as well is widely regarded as feminine especially by men with high conformity to TMI (45–47). Many men understand psychotherapy as something that is offered by women for women with the goal of emotional closure (48). Indeed, central qualities beneficial for the psychotherapeutic process such as accepting help, exposing vulnerability, or emotionality in therapy are antithetical to TMI of toughness, restrictive emotionality, self-reliance, and dominance and they introduce male-specific therapy interfering processes (16, 49, 50).

Thus, suffering from a depression and taking on psychotherapy clearly violates several TMI and creates gender role conflict, reflecting the psychological distress of men when behaving differently than endorsed TMI would require (40). This is supported by studies showing that as compared to women, men in general exhibit reduced outcome expectation at the beginning of psychotherapy for depression (51, 52) and show increased irritability, a tendency to overreact, exhibit more anger attacks and lower impulse control (53). Due to the resulting therapy-interfering processes associated with men’s gender role conflict (40), depressed men are less likely to self-disclose and more likely to self-stigmatize, to resist, reject, and to early terminate psychotherapy (49, 54, 55). The research presented above illustrates the potential of a male-specific psychotherapeutic program (MSPP) addressing gender role conflict early in therapy and being tailored to the needs of depressed men. Although many recommendations for adaptation for male-specific psychotherapy of depression have been derived (54, 56) and there were also promising approaches to male-specific therapy interventions aiming to solve gender role conflict and conduct gender role re-evaluations in male-only group therapies (57–60), these interventions have never been integrated and systematically evaluated. The present MSPP approach for MDD is based on cognitive behavior therapy (CBT), and further integrates central aspects of male gender role socialization, TMI, gender role conflict, and psychological defense mechanisms in response to masculinity challenges.

Furthermore, over the last two decades, a dramatic increase in testosterone prescriptions was observed (61), as studies have continuously confirmed the safety of testosterone therapies and further demonstrated their potential as a male-specific antidepressant (62–64). Importantly, men with hypogonadism, which is defined by testosterone concentrations below 12.1 nmoL/L in the blood, are at increased risk for depressive disorders (65), while men with depressive disorders are at increased risk for low testosterone concentrations (66). These findings provide an evidence-base for stratifying male depression patients based on their testosterone status. Depressed men with comorbid hypogonadism may therefore benefit from receiving a combination therapy that integrates psychotherapy and pharmacotherapy for optimal treatment.

### 1.1. Aim and research question

This study aims to evaluate the world’s first MSPP for MDD in the single-setting in depressed eugonadal and hypogonadal men receiving testosterone. The primary goal is to evaluate the potential superiority in efficacy (score reduction of ≥50% or greater) and acceptability (proportion of men who withdraw for any reason) of the MSPP in comparison to a standard CBT for MDD and a Waitlist. Primary outcomes are depression symptoms scores (self-reported [BDI-2-21, MDRS−22] and observer assessed [HDRS-21]) proportion of achieved remission (assessed by structured clinical interview [SCID-5]), drop-out, gender role conflict (GRCS-16), the working alliance quality (BPSR-P-22, WAI-SR), and changes in the Clinical Global Impression Scale (CGI-S/I). We hypothesize that the MSPP group shows a significantly larger improvement in treatment outcomes at the end of the treatment and at 3-month follow-up compared to the CBT and Waitlist groups. We further hypothesize that all three TT-groups (MSPP+TT, CBT+TT, Waitlist+TT) exhibit higher symptom reduction than the parallel eugonadal groups (MSPP+TT > MSPP, CBT+TT > CBT, Waitlist+TT > Waitlist) due to the TT-adjunct effect. We also expect that added psychotherapy to TT leads to a greater reduction of depression symptoms and that the MSPP achieves the largest symptom reduction (MSPP+TT > CBT+TT > Waitlist+TT).

Our second goal deals with psychological and physiological moderators and mediators of treatment outcome such as endorsement of traditional masculinity and precarious manhood beliefs (MRNI-SF-21, CMNI-SF-30, PMBS-4, GRDS-10), suicidality (SSEV-9, SCS-18), traumatization (CIDI-Trauma-12, ITQ-18), alcohol use (AUDIT-10), anxiety (GAD-7), sexual dysfunction (IIEF), anger and aggression (BP-AQ-36), body image (FKB-6, BAS-2, MBAS-R-15), self-stigma (STIG-9, SSOSH-10), shame, guilt and self-esteem (SHAME-21, TOSCA-16, RSES-10), emotion regulation skills (ERQ-10, SCS-D-12, LS-6), need to belong and general belongingness (NTB-10, GBS-12), and sensation seeking (AISS-D-20) (description and references of used psychometric instruments is provided in Supplementary Table S1). Further, we examine several physiological and biological markers (e.g., blood and hair testosterone, bioelectrical impedance analysis [BIA]) over the course of treatment with the goal to identify potential male-specific treatment-response predictors and treatment-sensitive biomarkers useful as treatment monitoring markers.

The third goal of this study is to investigate male-specific factors that are associated with an increased risk of developing MDD characterized either by prototypical or male-specific externalizing depression symptoms. To this end, group comparisons will be conducted between depressed men and healthy control men with regard to central constructs such as conformity to TMI, gender role discrepancy stress, and testosterone status.

## 2. Methods and analysis

### 2.1. Design

**Figure 1:**
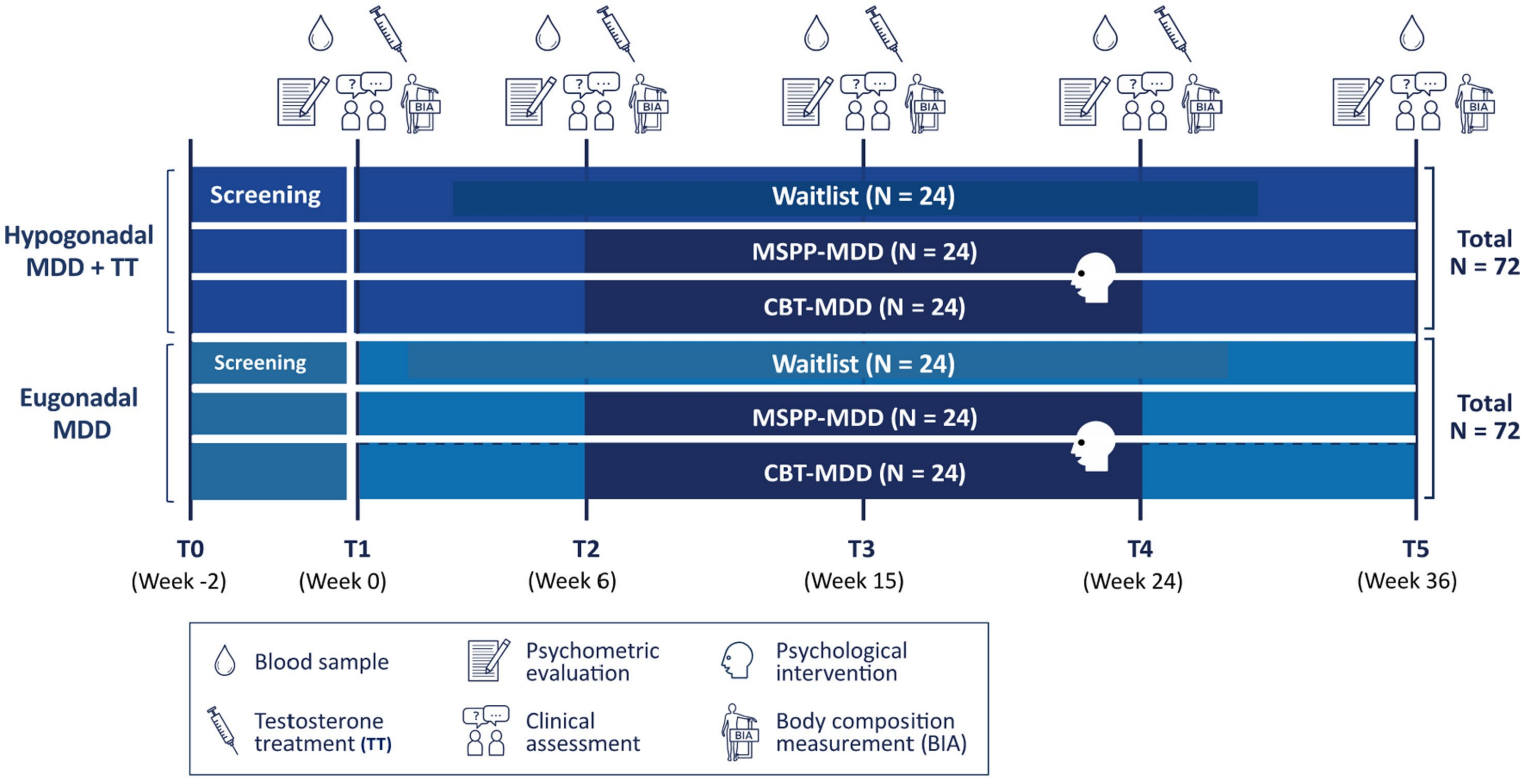
Six-arm randomized clinical superiority trial using a 2×3 factorial study design. MDD, major depressive disorder; T, timepoint; MSPP, male-specific psychotherapeutic program; CBT, cognitive behavioral therapy; N = group size.

The study is a six-arm randomized clinical superiority trial. Participants will be stratified by testosterone status (eugonadal or hypogonadal) and then randomized (stratified randomization) scheme by testosterone status combined with permuted block randomization (67, 68) into one of the three conditions (MSPP, CBT, or Waitlist). Thus, the study presents a 2×3 factorial study design as shown in Figure 1. In addition, a separate healthy control group will be examined, which will undergo only baseline assessments.

### 2.2. Sample and recruitment

One hundred and forty-four participants with the primary diagnosis of a MDD will be recruited. In addition, one hundred healthy control participants will be recruited, which will undergo only baseline assessments. Inclusion criteria are being cis-men (assigned male at birth and identifying as man), aged between 25 and 50 years, understanding and speaking sufficiently well German and for cases presence of a major depressive episode (assessed by structured clinical interview [SCID-5]). Exclusion criteria are inability to provide written informed consent, prior hormonal (e.g., testosterone) treatment, prior mental health disorder, current or previous psychopharmacological or psychological treatment, another current psychological disorder (e.g., bipolar disorder, anxiety disorders, etc.), a severe somatic disorder warranting priority treatment (e.g., cancer, neurological disorder, diagnosed prostate cancer), current treatment with specific medication types (e.g., thyroid hormones, finasteride), and specific genetic or hormonal disorders such as Klinefelter’s syndrome or Cushing’s or Addison’s disease. For the healthy control group, the same inclusion and exclusion criteria apply as described above, except for the depression diagnosis. Participants are only included as healthy controls if they do not have a major depression, but otherwise fit the inclusion and exclusion criteria. For the detailed description of inclusion and exclusion criteria see Table 1.

**Table 1:** Inclusion and exclusion criteria.

Inclusion criteria	
Male	
Age between 25 and 50 years	
German speaking	
Informed consent as documented by signature	
Current major depression (assessed by SCID-5) of mild or moderate degree.	
Exclusion criteria	
Inability to give informed consent	
Prior hormonal (testosterone) treatment	
Prior mental health disorder	
Current or previous psychopharmacological treatment	
Severe major depression with indication for combination treatment (psychopharmacotherapy and psychotherapy)	
Acute suicidality	
Current or previous psychological treatment for any psychological disorder	Prior use of outpatient psychotherapy with a psychological psychotherapist or psychiatrist, previous hospitalization in a psychiatric hospital
Comorbidities of major depression with any other psychological disorder^a^:	Bipolar disorder^b^ Psychotic symptoms^c^ Schizophrenia Anxiety disorder Obsessive compulsive disorder Post-traumatic stress disorder Attention deficit hyperactivity disorder Eating disorders Personality disorder Moderate or severe alcohol/substance abuse disorder (SCID-5, 4–11 criteria met)
Severe physical disorder that requires priority treatment such as:	Cancer Brain injury Severe neurological disease Multiple sclerosis Epilepsy Muscular dystrophy Obesity (BMI > 35 kg/m2)
Report of presence of any of the following physical conditions, particularly relevant in regard to testosterone treatment	Diagnosed prostate cancer Prostatic intraepithelial neoplasia (PIN) Severe lower urinary tract symptoms^d^ Erythrocytosis, defined as a hemoglobin >16.0 g/dL Sleep apnea, diagnosed but untreated
Current treatment with:	Thyroid hormones Finasteride Antiepileptic drugs Anabolic compounds Hypnotic medication more than 2 nights/week for the treatment of insomnia Long-acting benzodiazepines Antipsychotic medication Drugs that affect serum testosterone^e^
Genetic/hormonal disorders	Klinefelter’s syndrome Cushing’s disease Addison’s disease Hashimoto Thyroiditis

^a^Exception for persistent depressive disorder (Dysthymia) and alcohol/substance use disorder of mild degree (SCID-5, 1–3 criteria met). ^b^One manic episode is sufficient for exclusion. ^c^Including psychotic disorders and severe major depression with psychotic symptoms. ^d^Score of ≥ 19 by the International Prostate Symptom Score questionnaire. ^e^Specifically, use (during the previous 6 months) of drugs that affect the serum testosterone concentration, e.g., testosterone, androstenedione, dehydroepiandrosterone, estrogens, spironolactone, ketoconazole, and opiates.

Patient recruitment will be promoted by various psychotherapy institutes addressing men who are eligible (e.g., the Psychotherapeutic Center of the University of Zurich). In addition, media presence is used to advertise participation. Hypogonadal depressed men will additionally be recruited *via* Andrology/Urology Clinics and Centers (Uroviva Network). Healthy men are recruited through various channels. Media presence, social media advertising and snowball sampling is used to advertise participation. Men can apply to participate through an online portal called Andromind^©^ designed for psycho-educative purposes about men’s mental health. Therapists will be mainly recruited through the Psychotherapeutic Center of the University of Zurich. Therapists must be well advanced in training as psychological psychotherapist with a focus on cognitive-behavioral therapy (CBT).

### 2.3. Procedure

**Figure 2:**
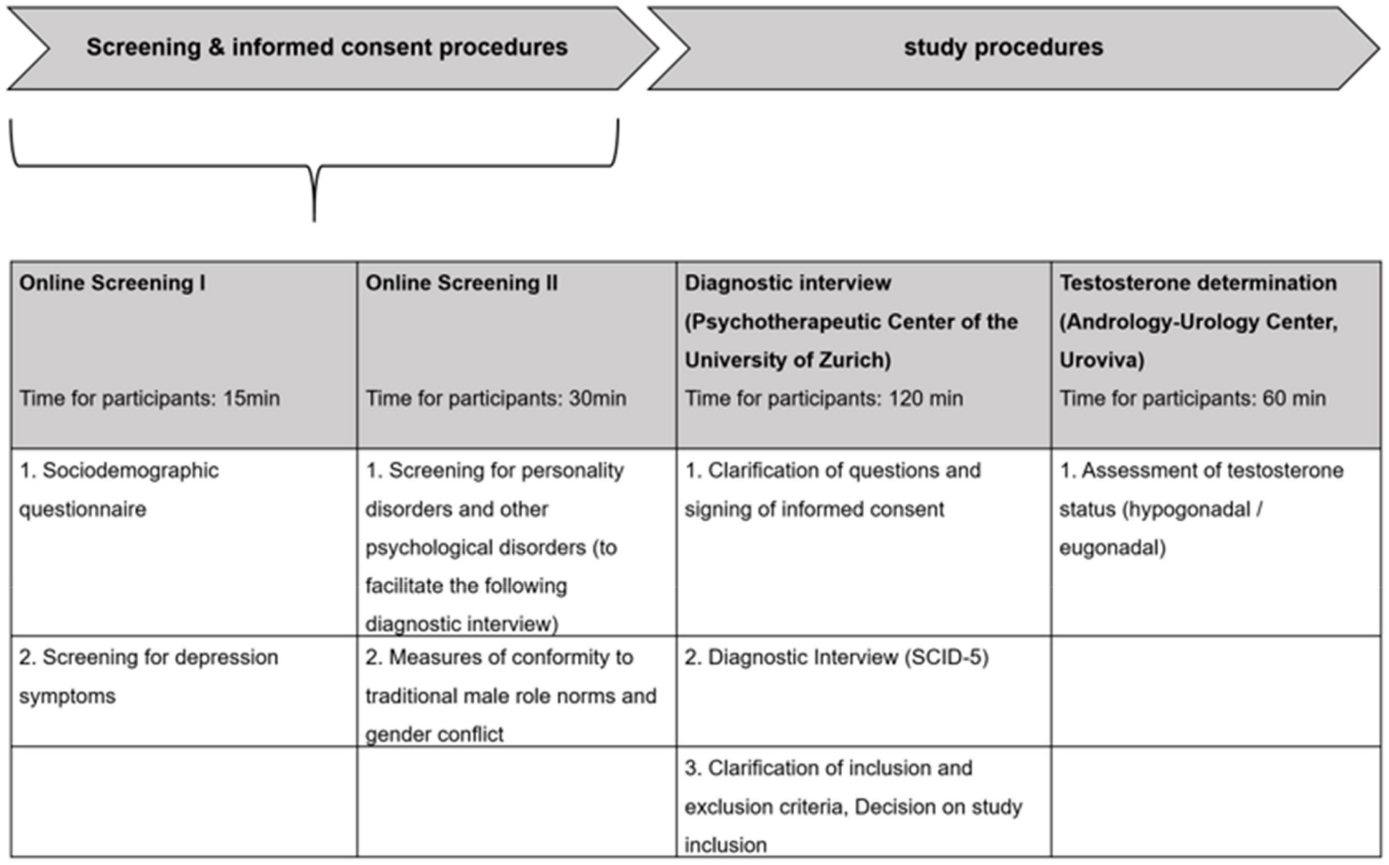
Screening and informed consent procedures.

Figure 2 gives an overview over the screening procedures. Interested men will complete an online-screening questionnaire assessing socio-demographic data and study eligibility (e.g., inclusion/exclusion criteria and depression symptoms). Depressive symptoms will be examined with the Patient Health Questionnaire-9 (PHQ-9) and the Male Depression Risk Scale-22 (MDRS-22; relevant cut-offs: PHQ-9 ≥ 10/MDRS-22 ≥ 51). In case of eligibility, participants are invited to a diagnostic interview at the Psychotherapeutic Center of the University of Zurich and are provided with study information, a consent form and a link to complete an additional psychometric test battery, screening for comorbidities such as personality disorders (SCID-5) to support and speed up the diagnostic interview.

At intake at the Psychotherapeutic Center, comprehensive information about the study is provided and open questions are explained carefully. Subsequently, inclusion and exclusion criteria are double checked and then written informed consent is obtained. Afterward, a structured clinical interview (SCID-5) is conducted in full to confirm a MDD diagnosis and rule out other psychological disorders.

Men who do not comply with the inclusion and exclusion criteria, will be advised that unfortunately they cannot further participate in the study but can seek help for their psychological distress *via* the Psychotherapeutic Center of the University of Zurich. Men for who a MDD in the clinical interview is confirmed and do comply with the inclusion and exclusion criteria will then be informed of the further study procedures and appointments will be made for testosterone determination with the Andrology-Urology Center (Uroviva).

Men who show a testosterone level greater than 12.1 nmoL/L in the first measurement (overnight fasting morning total testosterone) will then be stratified into the eugonadal group and randomized into either MSPP, CBT, or Waitlist. Men who have a level of 12.1 nmoL/L or less in the first testosterone measurement will be asked to come back for testosterone measurement 1 week later. If these men then show a level above 12.1 nmoL/L, they will also be assigned to the eugonadal group. If the testosterone level is for a second time confirmed to be below 12.1 nmoL/L, the men will be medically evaluated and if showing signs and symptoms of hypogonadism (e.g., reduced libido or erectile dysfunction) and being eligible for the study, they will be assigned to the hypogonadal group and randomized to one of the three conditions (MSPP+TT, CBT+TT, Waitlist+TT).

Once randomization has taken place, participants will be invited to the baseline examination (T1/Week 0) at the Psychological Institute of the University of Zurich. There, all primary and secondary observer-assessed and self-report outcome measures are collected, as well as a blood sample and hair sample, and a bioelectrical impedance analysis (BIA) is conducted. These measurements are repeated after 6, 15, 24, and 36 weeks as shown in Figure 1.

The active treatment phase lasts 18 weeks, while screening and lead-in phase last another 2 and 6 weeks. With the additional follow-up examination 12 weeks after the last session of active psychotherapeutic treatment, study participants will be in the study approximately for 38 weeks. Participants of the separate healthy control group will only partake in the first examination appointment (T1/Week 0). Thus, for participants of the healthy control group, the study duration is 2 weeks. Estimated duration for the main investigational plan is 34 months from start of screening of the first participant (01/2023) to the last participant visit and finishing of the study (10/2025).

The main incentive to participate in the study is the free-of-charge psychotherapy for all 144 RCT participants. Waitlist control participants will need to complete the waiting period of 36 weeks first. Furthermore, all men will get a free of charge testosterone status assessment and in case of hypogonadism and willingness state-of-the-art TT. Since dropouts with up to 25% are a major problem in psychotherapy studies with depressed men (69, 70), our study features several measures to support the compliance in the participants: a continuous personal contact to a therapist every week in active treatment groups, face-to-face diagnostic assessment at the beginning of the study by the study team, and continuous testing at weeks 0, 6, 15, 24, and 36 will be conducted for all men. Men in the Waitlist condition not receiving any form of treatment will be financially renumerated with 200.- Swiss Francs. Thus, the study includes several interventions to develop and maintain sustained motivation.

Blinding procedures: Participants are blinded to the received therapy modality (MSPP or CBT) and treating psychotherapists are not allowed to comment on the therapy modality. The blinding procedure regarding treatment condition (MSPP or CBT) is not applicable for therapists. After randomization, the provider will actively apply the treatment and thus the therapists as well as their supervisors will have knowledge about the patients’ treatment condition. Members of the study group are blinded to participants treatment condition (MSPP, CBT, or Waitlist). However, the study group is not blinded regarding gonadal status (hypogonadal or eugonadal) and TT. Likewise, blinding regarding testosterone status is not applicable for the TT delivering medical practitioner; however, he will also be blinded to patients’ treatment condition (MSPP, CBT, or Waitlist). Only an external randomization manager organizing the participant allocation is not blind to allocation condition but is not involved in diagnostics or assessment of the primary and secondary outcome measures.

### 2.4. Participant safety and monitoring

Although there are no indications that CBT or MSPP carry risks for participants with MDD, increase in suicidal ideation and psychotherapy specific (minimal) adverse risk factors will be monitored throughout treatment. As soon as TT is initiated monitoring of testosterone levels, relevant parameters (e.g., prostate specific antigen [PSA], hematocrit), side effects and adverse events will be conducted in routine clinical treatment by the TT supervising medical practitioner. In case abnormal results are observed (e.g., testosterone levels greater than 28.7 nmoL/L), the medical practitioner may consider lengthening the injection interval. However, if full blood count hemoglobin is greater than 18 g/dL, or hematocrit is greater than 0.54 L/L, or PSA is greater than 4ug/L, and there are concerns about the health of the study participant, the medical practitioner may discontinue the TT and evaluate the cause of abnormal parameter levels. Deviations from the routine TT procedure will be documented and at the end of the study period of 36 weeks, it will be evaluated whether these deviations lead to exclusion of the study participant. Psychotherapeutic treatment or Waitlist will continue unchanged.

### 2.5. Psychotherapies

All psychotherapies are carried out by clinical psychologists trained in CBT who are already working as case-leading psychotherapists under supervision in a mental health institution. Regular supervision by trained supervisors as well as video recordings of the therapy sessions guarantee manual adherence and therapeutic quality of the MSPP and CBT for MDD. All psychotherapists receive a training day in manualized MSPP, respectively, CBT for MDD. To prevent carryover effects, therapists provide either the MSPP or CBT. Each patient is accompanied and guided by one of 20 study psychotherapists.

Both, the MSPP and CBT comprise 18 sessions delivered over a period of 18 weeks with weekly sessions and homework bridging sessions. The development and structure of the MSPP is described below. The CBT is applied in its manualized form as described by Hautzinger (71).

### 2.6. Male-specific psychotherapeutic program for MDD (MSPP-MDD)

The present MSPP for adult MDD was designed to be used for the acute outpatient treatment of men suffering from MDD. The MSPP-MDD is based on the CBT-manual for depression treatment according to Hautzinger (71–74). Central CBT elements will be retained, while the entire MSPP will be embedded in a male gender conscious setting.

The general novelty of the MSPP-MDD lies in the cooperative work of the therapist and client to examine, acknowledge, and if necessary, modify the endorsement of TMI, to solve gender role conflicts, and to stabilize the challenged masculinity self-concept and self-worth. In the following, the therapeutic attitude, structural aspects, the standardized and systematic examination of gender role conflict, and male-specific adaptations in the MSPP-MDD based on CBT for MDD shall be briefly outlined.

#### 2.6.1. Therapeutic attitude

A supportive therapeutic attitude employing Socratic questioning and focusing on central problems is important to promote the therapeutic process. It is often the case that men are reserved and less emotionally accessible at the beginning of therapy than women. This is often due to the endorsement of TMI of for example status and dominance, which can be overcome by conscious self-disclosure techniques on the part of the therapist to show the male client that he has no reason to be skeptical by regaining perceived control and reduce status and dominance threats. As the Group for the Practice with Boys and Men suggests, therapists in the MSPP are trained to ask their male clients direct questions about mood and affect and to be prepared to ask more detailed questions in response to short answers (American Psychological Association Boys and Men Guidelines Group, 2018).

#### 2.6.2. Structural aspects

In brief, six empirically informed domains structured in six therapy phases comprising a total 18 therapy sessions à 50 min as described in more detail in Table 2 are tailored to men, while recognizing diverse client sensitivities that vary within and across the MSPP-MDD. Therapies will be free of charge due to their research purpose. In terms of literacy, the men’s language and understandings will be evaluated and integrated to derive how the intervention components are named and delivered. In terms of expectations and roles, the success of the MSPP-MDD will be explicitly positioned as contingent on both the work and commitment of the provider and client with clearly delineated individual and collaborative deliverables.

**Table 2:** Treatment plan of the male-specific psychotherapeutic program for acute major depression (MSPP-MDD) in the single setting compared to CBT-MDD^*^.

Phases	Therapy sessions	Single setting therapy (session time is 50 min)
I	1–2 (2)	- Establishment of a therapeutic relationship - Anamnesis - Life history interview - Safety planning/non-suicide agreement - Presentation of contingency plan and its application (information also provided as booklet) - Completion of a set of questionnaires as homework
II	3–5 (3)	- Problem and goal analysis - Explanation and psychoeducation of the nature of depressive disorders and their dynamic (cognition, behavior, emotion interconnectedness) - Introduction of typical depression spirals - Development of an individual disease model - Psychoeducation of male-typical expression of depression - Exercise for the cooperative identification of traditionally oriented strengths of men (e.g., generative fatherhood, male courage, humor, and heroism) - Psychoeducation of the relation between depression and gender role identity, endorsement of traditional masculinity ideologies, and gender role conflict - Standardized assessment of gender role conflict (O’Neil, 2013) - Psychoeducation of psychological defense mechanisms in response to masculinity challenges - Development of a personalized disease model including a gender perspective - Derivation of therapy structure and elements
III	6–9 (4)	- Behavioral activation - Structuring of everyday life - Week plan protocol - Reduction of stressful and increase of pleasant activities - Recognition of the connection between pleasant experiences and well-being - Focusing of build-up of activities that support the currently destabilized masculinity self-concept (e.g., sports)
IV	10–13 (4)	- Recognizing dysfunctional and functional cognitions - Identifying automatic negative thoughts - Day protocol of negative thoughts (with 3 and 5 columns) - Cognitive renaming - Recognizing and changing dysfunctional beliefs - Inclusion of the gender-conscious framework for cognitive techniques (e.g., cognitive restructuring of masculinity as independent of depression or psychotherapy) - Application of cognitive techniques to solve potential gender role conflicts
V	14–17 (4)	- Provision of skills fostering interpersonal relations (e.g., overcoming behavioral problems) - Practicing with role plays (social, interactive, problem-solving) - Behavioral exercises - Shaping relationships (involvement of partner, family, friends) - Conducting the Impact Massage Inventory - Improving interaction style with male-specific framework (e.g., improve the ratio between hostile-dominant interactions and friendly-dominant or friendly-submissive interactions)
VI	18 (1)	- Maintenance of progress through continuation of exercises - Recognizing crises, setbacks, and new episodes - Detection of early symptoms and control options - Contingency plan

^*^Table 2 presents an adapted treatment plan from Hautzinger (73) for the treatment of acute major depression with MSPP adjustments below the dashed line in the single setting therapy column. Impact Message Inventory refers to the Impact Message Inventory – Circumplex (IMI-C) by Kiesler et al. (2006) (76).

#### 2.6.3. Gender role conflict assessment

Gender role conflict (GRC) is often unconscious and rarely mentioned as a direct problem by clients. In the MSPP-MDD, the examination and discussion of GRC will be conducted early in therapy as part of psychoeducation about the nature of depressive disorders and their dynamics in order to frame it as a male-specific depression psychoeducation. This allows the therapist and client to refer back to them throughout therapy and quickly resolve therapy interfering processes grounded in GRC. Special attention shall be given to the standardized assessment of GRC as described by O’Neil (40, 41). The nine different manifestations of GRC can be assessed with standardized screening questions to examine the male clients’ GRC.

#### 2.6.4. Male-specific adaptations

The MSPP is structured around the established six phases of CBT for depression according to Hautzinger (71–73, 75). For comparison of a standard CBT-MDD with a MSPP-MDD, a session number of 18 sessions is chosen. This guarantees that, on the one hand, enough sessions are available to allow the different therapeutic effects of the two approaches to manifest themselves, while on the other hand, a restriction to 18 sessions does not allow any excesses in order to carry out the clearly manualized program. Subsequently, Table 2 presents the original and adapted six therapy phases of the CBT-MDD and MSPP-MDD. MSPP-MDD adjustments for each phase are listed below the gray line.

### 2.7. Waitlist condition

The participants in the Waitlist conditions (Waitlist alone in eugonadal men; Waitlist+TT in hypogonadal men) are required to complete a waiting period of 36 weeks prior receiving the MSPP-MDD. For all men in a Waitlist condition receiving no treatment for 36 weeks, a financial compensation of CHF 200.- will be provided with CHF 100.- funded after the third study examination visit at week 15 and the second CHF 100.- after completion of the last examination study visit at week 36. However, men in the Waitlist conditions will be monitored by means of three appointments at week 6, 10, and 14 with a study psychologist [previously termed contact control group (77)]. In these three 50-min sessions no intervention will occur, while a treatment relationship is to be established so that the patient can describe his specific life circumstances and report possible symptom exacerbations or suicidality. Psychotherapists are instructed to respond to patients with empathy and supportive reassurance without using specific interventions as in the MSPP or CBT conditions. However, if necessary—for example, if suicidality of the participant becomes apparent—the study psychologist immediately takes the necessary steps to ensure that the participant receives adequate treatment. Subsequently, monthly telephone calls at weeks 18, 22, 26, and 30 are conducted where the therapist inquiries about the patient’s condition and routinely assesses for suicidality. Having completed the follow-up assessment at week 36, Waitlist participants start with the psychotherapy. Since drop-out rates for Waitlist conditions in psychotherapy trials seem not increased in comparison to active groups (69, 78) and a 9-month waiting period for psychotherapy is not uncommon in general care, no additional compliance interventions are provided.

### 2.8. Testosterone treatment

Participants with diagnosed hypogonadism will be treated with TT for example in the cooperating Andrology-Urology Center (Uroviva). Participants start with testosterone undecanoate (NEBIDO®) at Week 0 after the baseline assessment. Testosterone undecanoate is administered *via* deep intramuscular injection, 1 g every 10–14 weeks. At the beginning a build-up of testosterone is conducted with a second injection after six weeks and following injections after 10–14 weeks (Figure 1). The TT for primary or secondary Hypogonadism will financially be covered by the participant’s own health insurance.

### 2.9. Material and measures

A wide array of different primary and secondary outcome measures is obtained. There are observer-assessed primary outcomes, as well as self-reported primary and secondary outcomes, video recordings of the therapy sessions, and physiological and biological parameters obtained. Table 3 provides an overview of all instruments and measures that will be used and obtained at specific examination time points, while also delineating assessments in the screening phase and dropout assessment. Correspondence between participants and therapists, worksheets and flip-charts created during the sessions will also be stored.

**Table 3:** List of primary and secondary outcome measures by study visit.

Study visit	Outcome measure	Time (min)	Examiner	Online screen I & II (T0a)	Diagnostic interview (T0b)	Testosterone status (T0c)	T1	T2	T3	T4	T5	Drop-out assessment
Weeks				−2	−2	−2/−1	0	6	15	24	36	
Informed consent, demographics	S	5	*C*	x								
checking study in−/exclusion criteria/screening for depression and hypogonadism	S	10	*C*	x								
Screening for personality disorders and TMI	S	30	*C*	x								
Structured Clinical Interview for DSM-5 (SCID-5 complete version/only MDD at T4)	S/PO	60/15	*R*		x					x		x
Screening for problematic internet use, gaming disorder, narcissism, childhood trauma	S	10	*C*		x							
Hypogonadism assessment (total testosterone blood)		60	*M*			x						
Observer assessed primary outcome measures												
Hamilton Depression Rating Scale (HDRS-21) Clinical Global impression Scale CGI-S/I	PO	15	*R* *R*				x	x	x	x	x	x
Self-rated primary outcome measures												
Male Depression Risk Scale (MDRS-22) Beck Depression Inventory II (BDI-2-21) Gender Role Conflict Scale (GRCS-16) Working alliance quality (BPSR-P-22, only at T3, T4, T5)	PO	15	*C*				x	x	x	x	x	x
Self-rated secondary outcome measures (I)												
Skala Suizidales Erleben und Verhalten (SSEV-9) Suicide Cognitions Scales (SCS-18) Alcohol Use Disorder Test (AUDIT-10) Problematic Pornography Consumption Scale (PPCS-18) International Index of Erectile Function (IIEF-15) Perceived Stress Scale (PSS-10) Generalized Anxiety Disorder (GAD-7) Conformity Masculine Norms Inventory (CMNI-30) Male Role Norms Inventory (MRNI-SF-21) Precarious Manhood Beliefs Scale (PMB-4) Gender Role Discrepancy Stress (GRDS-10) Loneliness Scale (LS-20) Self-Compassion Scale D (SCS-D 10) Emotion Regulation Questionnaire (ERQ-10) General Belongingness Scale (GBS-12)	SO	30	*C*				x	x	x	x	x	
Self-rated secondary outcome measures (II)												
CIDI-Traumaliste (CIDI-T-12) International Trauma Questionnaire (ITQ-18) Buss-Perry Aggression Questionnaire (BPAQ-36) Fragebogen zum Körperbild (FKB-6) Male Body Attitudes Scale–Revised (MBAS-R-15) Body Appreciation Scale 2 (BAS-2-10) Stigma questionnaire (STIG-9) Self-stigma of seeking psychological help (SSOSH-10) SHAME Questionnaire (SHAME-21) Test of Self-Conscious Affect (TOSCA-16) Toronto Alexithymia Scale (TAS-20) Rosenberg Self-Esteem Scale (RSES-10) Arnett Inventory of Sensation Seeking (AISS-20) Need to Belong Scale (NTBS-10)	SO	35	*C*				x	x		x	x	
Qualitative and adverse events measures												
Qualitative semi-structured interview (treatment experience)	SO	25	*R*							x		x
Checklist of adverse events	SO	5	*R*						x	x	x	x
Samples and procedures for biomarker analysis												
Body composition (BIA)	SO	5	*N*				x	x	x	x	x	
Blood (50 mL), Blood pressure	SO	10	*N*				x	x	x	x	x	
Hair sampling (10 mg)	SO	5	*N*				x	x	x	x	x	
Grip strength	SO	2	*N*				x	x	x	x	x	
Subjective psychotherapy process measures												
Bern Post Session Assessment (BPSR-P-22, WAI-SR) – Patients Bern Post Session Assessment (BPSR-T-27, WAI-SR) – Therapist	EA	5	*C*									
Objective psychotherapy process measures												
Video recording of psychotherapeutic sessions	EA	-	*T*									
Time requirement for patient only (min)				45	120	60	120	120	85	160	120	75

S, screening; M, medical Doctor; C, computerized assessment; R, research psychologist (observer ratings); N, study nurse; T, therapist; PO, primary outcome measure; SO, secondary outcome measure. TMI, traditional masculinity ideology (CMNI-30, MRNI-21, PMB-4, GRCS-16); EA, exploratory analysis. Self-rated secondary outcome measures (I): assessed at every examination visit, Self-rated secondary outcome measures (II): assessed at T1, T4, T4, Time requirement are calculated with enough time considering that depressed individuals take longer for completion of psychometrics and responding to clinical assessment. Observer assessed primary outcome measures: Total item number = 23, Self-rated primary outcome measures: Total item number = 81, Self-rated secondary outcome measures (I): Total item number = 176, Self-rated secondary outcome measures (II): Total item number = 22. Columns in light red represent the screening and diagnostics period, columns in green represent the trial period, columns in grey represent the dropout period.

### 2.10. Data analysis

Relevant participant characteristics will be reported at baseline measurement for each stratum separately (i.e., hypogonadal and eugonadal testosterone status). To assess potential differences in continuous outcomes fulfilling conditional normality and homoscedasticity constraints, Student’s two-sample t-test will be used. For heteroscedastic outcomes, a Welch-Satterthwaite approximation to the degrees of freedom will be used (79). If conditional normality is not present, the nonparametric Wilcoxon rank-sum test (80, 81) will be applied instead. To assess potential dependencies in categorical data with expected counts larger than five in all categories, Pearson’s Chi-squared test will be used. Alternatively, if this assumption is not met, a Monte Carlo simulation [*n*_rep._ = 2000 (80, 82);] on conditional marginal distributions will be applied.

To investigate the superiority hypotheses specified under the primary goal, three different multilevel models will be used to model the primary outcomes. The first model will contain the pooled parallel treatment groups (MSPP, CBT, Waitlist, irrespective of gonadal status) and time (linear) as fixed effects and random intercepts. The second model will be identical to the first model but include an additional interaction effect between the treatment group (MSPP, CBT, and Waitlist) and gonadal status (hypogonadal vs. eugonadal), thereby assessing treatment effects conditional on gonadal status. The third model will use a linear discontinuity approach to assess improvement over the course of the treatment (T2–T4) and into the follow-up (T4–T5) separately. Discontinuity is specified at T4 and treatment group (MSPP, CBT, and Waitlist) as well as time (linear) will be included as fixed effects in addition to random intercepts and slopes.

For the explorative investigation of the secondary and tertiary goal of this study, similar multilevel modeling approaches will be used as for the primary goal. Additionally, structural equations modeling will be used to assess potentially mediating effects of the secondary outcome variables. For baseline cross-sectional case control comparisons, t-tests will be used. The 100 healthy controls will be compared to the 144 depressed men, while when testosterone status will be included as additional grouping factor, ANOVA’s will be used.

#### 2.10.1. Power analysis

To identify differences for the reduction of depressive symptoms (effectiveness: significant difference in MADRS or MDRS; efficacy: 50% symptom reduction in depression score [positive/negative]) between the six groups (depending on three treatment groups and gonadal status [3×2 = 6]) based on repeated measures analysis including the interaction between treatment and time in the model and assuming a medium effect size (Cohen’s *f* = 0.25), excellent power (0.95) is reached with a sample size of 216.6. However, an acceptable power (0.80) would be achieved with a sample size of 142.5. When examining only treatment effects irrespective of gonadal status, a sample of 142.5 is associated with excellent power (>0.90). Assuming equal group number, a sample size of 144 (6×24) represents a good powered sample to detect effects that are of moderate size or greater. In case there are recruitment, participation, or dropout problems, groups containing 18 individuals (*n*_total_ = 108) would still provide acceptable power for the identification of treatment differences in symptom reduction.

For baseline group comparisons stratified by gonadal status resulting in three groups (healthy controls [HC], depressed eugonadal [MDD-E], and depressed hypogonadal [MDD-H]), ANOVAs will be calculated. For the identification of medium effect sizes (*f* = 0.25) and using an alpha error probability of 0.05, and a power of 0.90 a total sample of 207 would be required. However, the total sample of 244 including all groups would allow for the detection of small effect sizes with an acceptable power of 0.80.

Regarding the healthy control group, for baseline group comparisons, t-tests for the analysis of differences between two independent means will be calculated. For the identification of medium effect sizes (d = 0.5) and using an alpha error probability of 0.05, a power of 0.90, and an allocation ratio of 1, a total sample of 172 would be required. However, the total sample of 244 would allow for the detection of smaller effect sizes until d = 0.35 with an acceptable power of 0.80.

#### 2.10.2. Missing data

Different techniques for handling missing data may be considered, including complete case analysis, single imputation with either the mean or the last observation carried forward [LOCF; (83)], and multiple imputation with k-nearest neighbors [KNN; (84)]. The robustness of these analyses may be used to make a final decision on how to treat missing datapoints. A robust method is characterized by providing point estimates that are close to the complete case analysis and small confidence intervals.

### 2.11. Ethical considerations

Ethics approval was given by the Ethics Committee of the canton Zurich (Switzerland). Project-ID: 2022–01141. All participants will be informed in accordance with the study protocol approved by the Ethics Committee (Clinical Study Protocol Version 3, 15.12.2022).

An internal and external data and safety monitoring will be established to monitor all aspects of the study. The monitoring consists of an internal monitoring by a certified clinical trial manager. Furthermore, an external team from the Clinical Trials Center of the University Hospital of Zurich ensures data quality, progress of the study and patient safety. Whether the internal nor the external monitoring members will be affiliated in any way with the study.

## 3. Discussion

For MDD, there continue to be major difficulties in improving its treatment with only half of all clients responding to the evidence-based and recommended CBT treatment (85). The field of psychotherapy is therefore increasingly aiming toward personalization of therapies for patients. There is a growing consideration of gender-sensitive approaches in both pharmacological and psychotherapeutic research (56, 63, 86). The proposed project evaluating the potential superiority of a male-specific CBT-based treatment, namely the MSPP, compared to standard CBT and Waitlist is the world’s first attempt to establish a standardized gender-specific psychotherapeutic program for men suffering from MDD in the single setting.

We therefore hypothesize that compared to the Waitlist control groups, the treatment groups will be more effective and efficacious (depression score reduction of ≥50%) at week 24 and at the follow-up at week 36. It further is hypothesized that the MSPP, compared to CBT, shows higher effectiveness and efficacy for depressive symptomatology (BDI-2, HDRS-21, SCID-5), clinical global impression (CGI), higher acceptability (lower dropout rate), a better working alliance quality (BPSR-P-22), and a greater reduction of gender role conflict (GRCS-16) as well as related TMI measures (CMNI-SF-30, MRNI-SF-21, PMB-4, GRDS-10).

The 2×3 factorial study design represents a very pioneering and state-of-the-art way to randomize male depressed psychotherapy patients according to their gonadal status. The methods used in the present study are in accordance with the gold standard of diagnostics (SCID-5), psychotherapy research (CBT), hypogonadism treatment (testosterone treatment with NEBIDO^®^), and is oriented toward patient well-being (contingency plans and continuous monitoring of depression symptoms and suicidality).

Regarding TT, we decided using testosterone injections applied by the TT medical practitioner instead of using testosterone gels for self-application by the participants. The rational for this lies within the control of application. Since compliance in depressed men with treatments is often insufficient, testosterone injections provide the best method to ensure reliable and standardized TT.

Prior psychological or pharmacological treatment, as well as psychological and physiological comorbidities may have a substantial impact on treatment response. That is why we decided to investigate the effects of the MSPP in first episode treatment naive men. Although it must be considered that the many inclusion and exclusion criteria, such as “no prior history of mental disorders” or “no current or previous psychotherapy use” may limit the generalizability of the study results. Furthermore, there is the risk, that the protocol might result being more time-consuming than the authors initially predicted. We acknowledge that the study presents a large measure protocol, which by itself harbors challenges. Should participants indicate that the protocol is to extensive and start to dropout in higher numbers due to the extensive measurement protocol become apparent, we will dynamically adjust the protocol to fit the resources of the participants. This is particularly important due to the specific characteristics of the study population, namely men with major depressive disorder. Likewise, if it turns out that depressed hypogonadal men are not as prevalent as originally thought and not enough hypogonadal men can be included into the RCT, the initially planned analyses for the eugonadal group alone will be performed and exploratory analyses for the group with hypogonadism will be conducted.

Due to the large body of research suggesting the male population and particularly the male population high in traditional masculinity to represent a challenging client group for psychotherapy, many researchers have written on the topic and made several recommendations for counseling men (40, 46, 50, 87, 88). However, besides male-tailored online resources (89, 90), only two attempts exist evaluating a male-specific form of psychotherapy treatment for mood disorders.

For example, in their pilot trial evaluating only five men, Primack et al. (2010) investigated a male-specific CBT-based psychotherapy for depression, named the “Men’s Stress Workshop.” Five men aged 38 or older completed at least seven of the eight treatment sessions or the intervention. A reduction in depression symptoms, an increase in social support connections, but also an increase in self-stigma was reported. Qualitative analysis of the men’s feedback to the intervention revealed that the focus on men was listed as the top reason for joining the workshop and the discussion of TMI was described as being the most helpful part of the workshop (59). Following Primack et al. (59), the MSPP will also provide information about male-specific depression peculiarities and the impact of male gender role socialization, TMI, and gender role conflict. The systematized male-specific adaptations will directly be compared to CBT for MDD and Waitlist, thereby enabling to dissect the effect of tailoring a CBT-based psychotherapy specifically to men and complementing it with specific interventions having been shown to be important in the treatment of depressed men (see Table 2).

Another study compared a gender-focused group psychotherapy including a gender-role re-evaluation component following a value-based Integrity model (57) to a non-gender-focused group psychotherapy also following a value-based Integrity model for recently separated men (58). Emotional expression and psychological well-being were improved in both groups, but gender role conflict did not change and no group differences were identified. However, it should be pointed out here that the male participants often had relatively little psychological distress. This reduces the statistical power to detect changes in the outcome measures, whereas in the present study, only men with a confirmed diagnosis of MDD are included. Furthermore, a ten-week group program is a very short period to reduce severe psychological distress and resolve gender role conflicts, why in the present study, an active treatment period of 18 weeks was chosen to ensure that the therapeutic effects of the MSPP are reflected at the symptom level.

### 3.1. Clinical implications

Public campaigns and other initiatives are trying hard to increase the psychotherapy uptake of men with mental health issues (91), while psychotherapy itself is not specifically adapted to men, which leads to the fact of continuously low rates of men engaging in psychotherapy (26). Therefore, a male-specific depression therapy may not only improve treatment outcomes in men who would have had psychotherapy anyway, but, due to the male-specific focus, many more men would be interested in psychotherapy for MDD because it is specifically targeted at them. By providing a male-tailored, efficacious, and acceptable psychotherapy program for MDD, a general increase in the number of men who take on psychotherapy would be the consequence increasing the number of men successfully engaging in psychotherapy and ultimately leading to reduced depressive burden in many men and thereby preventing many acts of suicide.

Successfully introducing psychotherapy in a particularly vulnerable group of men, namely hypogonadal depressed men, would furthermore open new avenues for joint treatment programs in several subgroups of vulnerable men. Never before was it considered that men suffering from hypogonadism would benefit from adjunct psychotherapeutic support. The MSPP may be better equipped than CBT to resolve existing gender role conflict related to hypogonadism in depressed men further demonstrating the high relevance and direct clinical impact of the presented study.

## Trial status

The study protocol has been approved by the Ethics Committee of the Canton of Zurich on [20.12.2022] (Number 2022–01141).

Trial registration was completed on [27.06.2022].

Recruitment started on [16.01.2023] and will presumptively end on [31.02.2025].

Protocol version number: 3. Protocol version date: 15.12.2022.

## Author contributions

AW, UE, MS, and DZ: design. AW: funding. AW, UE, MS, LE, and CF: writing. AW, MS, LE, UE, CF, NK, AH, TR, ZS, SR, DK, JSO, JLO, RW, and DZ: revision. All authors contributed to the article and approved the submitted version.

## Funding

This project was funded by the Swiss National Science Foundation—Ambizione (Project number: PZPGP1_201757) awarded to AW. The project was further supported by a grant from the Stiftung für Urologische Forschung (Zürich, Schweiz) awarded to AW.

## Conflict of interest

The authors declare that the research was conducted in the absence of any commercial or financial relationships that could be construed as a potential conflict of interest.

## Publisher’s note

All claims expressed in this article are solely those of the authors and do not necessarily represent those of their affiliated organizations, or those of the publisher, the editors and the reviewers. Any product that may be evaluated in this article, or claim that may be made by its manufacturer, is not guaranteed or endorsed by the publisher.
