# Supplementary material for: Evaluation of a male-specific psychotherapeutic program for major depressive disorder compared to cognitive behavioral therapy and waitlist: study protocol for a six-arm randomized clinical superiority trial examining depressed eugonadal and hypogonadal men receiving testosterone
Source: Front Psychiatry. 2023 Jun 21;14:1129386. doi: 10.3389/fpsyt.2023.1129386 (PMC10321526; doi:10.3389/fpsyt.2023.1129386)
Supplement: Supplementary file 1 [file Data_Sheet_1.PDF]

Supplementary Material

Evaluation of a male-specific psychotherapeutic program for major depressive disorder compared to cognitive behavioral therapy and waitlist: Study protocol for a six-arm randomized clinical superiority trial examining depressed eugonadal and hypogonadal men receiving testosterone

Walther, A. *, Ehlert, U., Schneeberger, M., Eggenberger, L., Flückiger, C., Komlenac, N., Heald, A., Rice, T., Palm, S.S., Seidler, Z., Ogrodniczuk, J., Oliffe, J., Rice, S., Kealy, D., Weber, R. , & Zimmermann, D.

* **Correspondence:** Dr. phil. Andreas Walther: a.walther@psychologie.uzh

1 Supplementary Tables

Supplementary Table 1. Description and references of used psychometric instruments

Measure	Original Reference	German Validation
**Screening measures**		
Patient Health Questionnaire 9 (PHQ-9)	Kroenke et al. (2001)	Gräfe et al. (2004)
Male Depression Risk Scale (MDRS-22)*	Rice et al. (2013)	Walther et al. (2021)
Aging Male Symptoms Rating Scale (AMS)	Heinemann et al. (1999)	
International Prostate Symptom Score (IPSS)	Barry et al. (1992)	Badia et al. (1997)
Super Brief Pathological Narcisism Inventory (SB-PNI)	Pincus et al. (2009) Schoenleber et al. (2015)	Morf et al. (2017)
Conformity to Masculine Norms Inventory (CMNI-30)**	Levant et al. (2020)	
Male Role Norms Inventory (MRNI-SF-21)**	Levant & Hall (2013)	Komlenac & Hochleitner (2021)
Gender Role Conflict Scale (GRCS-16)*	O'Neil et al. (1986)	Komlenac et al. (2018)
Precarious Manhood Beliefs Scale (PMB-4)**	Bosson et al. (2021)	
Gender Role Discrepancy Stress (GRDS-	Reidy et al. (2014)	

10)**	Reidy et al. (2016)	
Childhood Trauma Questionnaire (CTQ)	Bernstein et al. (1994)	Klinitzke et al. (2001)
Internet Gaming Disorder Questionnaire (IGDQ)	Lemmens et al. (2015)	Jeromin et al. (2016)
The Problematic and Risky Internet Use Screening Scale (PRIUSS)	Jelenchick et al. (2014)	
Bergen Social Media Addiction Scale (BSMAS)	Andreassen et al. (2012)<br>Andreassen et al. (2016)	
Structured clinical interview for DSM-5 disorders – clinical version (SCID-5-CV)	First et al. (2016)	Beesdo-Baum et al. (2019)
Structured clinical interview for DSM-5 personality disorder (SCID-5-PD)	First et al. (2016)	Beesdo-Baum et al. (2019)
Marlowe-Crowne Social Desirability Scale (MCSDS-10)	Crowne & Marlow (1960)	Stöber (1999)
**Observer assessed primary outcome measures**		
Hamilton Depression Rating Scale (HDRS-21)	Hamilton (1960)	Collegium Internationale Psychiatriae Scalarum (CIPS) (2005)
Clinical Global impression Scale (CGI-S/I)	Guy (1976)	Billen et al. (2020)
**Self-rated primary outcome measures**		
Male Depression Risk Scale (MDRS-22)*	Rice et al. (2013)	Walther et al. (2021)
Beck Depression Inventory II (BDI-2-21)	Beck et al. (1996)	Kühner et al. (2007)
Gender Role Conflict Scale (GRCS-16)*	O'Neil et al. (1986)	Komlenac et al. (2018)
Working Alliance Inventory (WAI-SR)	Hatcher & Gillaspy (2006)	Wilmers et al. (2008)
Bern Post Session Assessment (BPSR-P-22)	Flückiger et al. (2010)	
**Self-rated secondary outcome measures (I)**		
Conformity to Masculine Norms Inventory (CMNI-30)**	Levant et al. (2020)	
Male Role Norms Inventory (MRNI-SF-21)**	Levant & Hall (2013)	Komlenac & Hochleitner (2021)
Precarious Manhood Beliefs Scale (PMB-4)**	Bosson et al. (2021)	
Gender Role Discrepancy Stress (GRDS-10)**	Reidy et al. (2014)<br>Reidy et al. (2016)	
Skala Suizidales Erleben und Verhalten (SSEV-9)	Teismann et al. (2017)	

Suicide Cognitions Scale (SCS-18)	Bryan & Harris (2014)	Spangenberg et al. (2019)
Alcohol Use Disorder Test (AUDIT-10)	Burns (2010)<br>Saunders et al. (1993)	Dybek et al. (2006)<br>Rumpf et al. (2002)
Problematic Pornography Consumption Scale (PPCS-18)	Bőthe et al. (2018)	
International Index of Erectile Function (IIEF-15)	Rosen et al. (1997)	Wiltink et al. (2003)
Perceived Stress Scale (PSS-10)	Cohen et al. (1983)	Klein et al. (2016)
Generalized Anxiety Disorder (GAD-7)	Spitzer et al. (2006)	Löwe et al. (2008)
Loneliness Scale (LS-20)	Russell (1980)<br>Russell (1996)	Döring (1993)
Self-Compassion Scale D (SCS-D 12)	Raes et al. (2011)	Hupfeld & Ruffieux (2011)
Emotion Regulation Questionnaire (ERQ-10)	Gross & John (2003)	Alber & Kessler (2009)
General Belongingness Scale (GBS-12)	Malone et al. (2012)	
**Self-rated secondary outcome measures (II)**		
CIDI-Traumaliste (CIDI-T-12)	Wittchen & Pfister (1997)	
International Trauma Questionnaire (ITQ-18)	Cloitre et al. (2018)	
Buss-Perry Aggression Questionnaire (BPAQ-29) and 7 Questions regarding domestic violence	Buss & Perry (1992)	Herzberg (2003)<br>Werner & von Collani (2014)
Fragebogen zum Körperbild (FKB-6)	Clement & Löwe (1996)	Albani et al. (2006)
Male Body Attitudes Scale–Revised (MBAS-R-15)	Tylka et al. (2005)	
Body Appreciation Scale 2 (BAS-2-10)	Tylka & Wood-Barcalow (2015)	Behrend & Warschburger (2022)
Stigma questionnaire (STIG-9)	Gierk et al. (2018)	
Self-stigma of seeking psychological help (SSOSH-10)	Vogel et al. (2006)	
SHAME Questionnaire (SHAME-21)	Scheel et al. (2013)	Scheel et al. (2020)
Test of Self-Conscious Affect (TOSCA-16)	Tangney (1989)	Kocherscheidt et al. (2002)
Toronto Alexithymia Scale (TAS-20)	Bagby et al. (1994)	Franz et al. (2008)<br>Popp et al. (2008)
Rosenberg Self-Esteem Scale (RSES-19)	Fleming & Courtney (1984)	Von Collani & Herzberg (2003)

Arnett Inventory of Sensation Seeking (AISS-20)	Arnett (1994)	Roth (2003)
Need to Belong Scale (NTBS-10)	Leary et al. (2013)	
**Biological outcome measures**		
Body composition (BIA)		
Blood (50ml), Blood pressure		
Hair sampling (10mg)		
Grip strength		
**Qualitative and adverse events measures**		
Qualitative semi-structured interview (treatment experience)		
Checklist of adverse events		
**Subjective psychotherapy process measures**		
Bern Post Session Assessment (BPSR-P-22, WAI-SR) – Patients	Flückiger et al. (2010)	
Bern Post Session Assessment (BPSR-T-27) – Therapist	Flückiger et al. (2010)	
Working Alliance Inventory (WAI-SR)	Hatcher & Gillaspy (2006)	Wilmers et al. (2008)

2 References

Abler, B., & Kessler, H. (2009). Emotion Regulation Questionnaire – Eine deutschsprachige Fassung des ERQ von Gross und John. *Diagnostica*, *55*(3), 144–152. https://doi.org/10.1026/0012-1924.55.3.144

Albani, C., Blaser, G., Geyer, M., Daig, I., Schmutzer, G., Bailer, H., Grulke, N., & Brähler, E. (n.d.). *Überprüfung und Normierung des „Fragebogen zum Körperbild" (FKB-20) von Clement und Löwe (1996) an einer repräsentativen deutschen Bevölkerungsstichprobe*. *Z Med Psychol 15,* 99–109.

Andreassen, C. S., Torsheim, T., Brunborg, G. S., & Pallesen, S. (2012). Development of a Facebook Addiction Scale. *Psychological Reports*, *110*, 501–517. http://dx.doi.org/10.2466/02.09.18.PR0.110.2.501-517

Andreassen, C. S., Billieux, J., Griffiths, M. D., Kuss, D. J., Demetrovics, Z., Mazzoni, E., & Pallesen, S. (2016). The relationship between addictive use of social media and video games and symptoms of psychiatric disorders: A large-scale cross-sectional study. *Psychology of Addictive Behaviors*, *30*(2), 252–262. https://doi.org/10.1037/adb0000160

Arnett, J. (1994). Sensation seeking: A new conceptualization and a new scale. *Personality and Individual Differences*, *16*(2), 289–296. https://doi.org/10.1016/0191-8869(94)90165-1

Babor, T.F., de la Fuente, J.R., Saunders, J. et al. (1989). The Alcohol Use Disorders Identification Test: guidelines for use in primary health care. World Health Organization: Division of Mental Health. Geneva.

Badia, X., Garcia-Losa, M., Dal-Ré, R. (1997). Ten-Language Translation and Harmonization of the International Prostate Symptom Score: Developing a Methodology for Multinational Clinical Trials. *Eur Urol,* 31, 29–140. https://doi.org/10.1159/000474438

Bagby, R. M., Taylor, G. J., & Parker, J. D. A. (1994). The twenty-item Toronto Alexithymia scale—II. Convergent, discriminant, and concurrent validity. *Journal of Psychosomatic Research*, *38*(1), 33–40. https://doi.org/10.1016/0022-3999(94)90006-X

Barry, M. J., Fowler, F. J., Jr, O'Leary, M. P., Bruskewitz, R. C., Holtgrewe, H. L., Mebust, W. K., & Cockett, A. T. (1992). The American Urological Association symptom index for benign prostatic hyperplasia. The Measurement Committee of the American Urological Association. *The Journal of urology*, *148*(5), 1549–1564. https://doi.org/10.1016/s0022-5347(17)36966-5

Beck, A. T., Steer, R. A., & Brown, G. (1996). *Beck Depression Inventory–II (BDI-II)* [Database record]. APA PsycTests. https://doi.org/10.1037/t00742-000

Beesdo-Baum, K., Zaudig, M., & Wittchen, H.-U. (Eds.) (2019). SCID-5-CV: *Strukturiertes Klinisches Interview für DSM-5 Störungen – Klinische Version*. [Structured clinical interview for DSM-5 disorders – clinical version]. Hogrefe.

Beesdo-Baum, K., Zaudig, M., & Wittchen, H.-U. (Eds.) (2019). SCID-5-PD: *Strukturiertes Klinisches Interview für DSM-5 – Persönlichkeitsstörungen*. [Structured clinical interview for DSM-5 personality disorders]. Hogrefe.

Behrend, N., & Warschburger, P. (2022). Validation of a German version of the Body Appreciation Scale2 (BAS-2). *Body Image*, *41*, 216-224. https://doi.org/10.1016/j.bodyim.2022.01.020

Bernstein, D. P., Fink, L., Handelsman, L., Foote, J., Lovejoy, M., Wenzel, K., Sapareto, E., & Ruggiero, J. (1994). Initial reliability and validity of a new retrospective measure of child abuse and neglect. *The American Journal of Psychiatry, 151*(8), 1132–1136. https://doi.org/10.1176/ajp.151.8.1132

Billen, C., Schulte-Ostermann, M.A. & Huchzermeier, C. (2020). Clinical Global Impression – Corrections (CGI-C) – deutsche Übersetzung. *Forens Psychiatr Psychol Kriminol*, 14, 328–335. https://doi.org/10.1007/s11757-020-00599-9

Bosson, J. K., Jurek, P., Vandello, J. A., Kosakowska-Berezecka, N., Olech, M., Besta, T., Bender, M., Hoorens, V., Becker, M., Timur Sevincer, A., Best, D. L., Safdar, S., Włodarczyk, A., Zawisza, M., Żadkowska, M., Abuhamdeh, S., Badu Agyemang, C., Akbaş, G., Albayrak-Aydemir, N., … Žukauskienė, R. (2021). Psychometric Properties and Correlates of Precarious Manhood Beliefs in 62 Nations. *Journal of Cross-Cultural Psychology*, *52*(3), 231–258. https://doi.org/10.1177/0022022121997997

Bőthe, B., Tóth-Király, I., Zsila, Á., Griffiths, M. D., Demetrovics, Z., & Orosz, G. (2018). The Development of the Problematic Pornography Consumption Scale (PPCS). *The Journal of Sex Research*, *55*(3), 395–406. https://doi.org/10.1080/00224499.2017.1291798

Burns, E., Gray, R., & Smith, L. A. (2010). Brief screening questionnaires to identify problem drinking during pregnancy: A systematic review: Prenatal screening for alcohol use. *Addiction*, *105*(4), 601–614. https://doi.org/10.1111/j.1360-0443.2009.02842.x

Bush, K. (1998). The AUDIT Alcohol Consumption Questions (AUDIT-C) An Effective Brief Screening Test for Problem Drinking. *Archives of Internal Medicine*, *158*(16), 1789. https://doi.org/10.1001/archinte.158.16.1789

Buss, A. H., & Perry, M. (1992). The Aggression Questionnaire. *Journal of Personality and Social Psychology, 63*(3), 452–459. https://doi.org/10.1037/0022-3514.63.3.452

Bryan, C.J. & Harris, J.A. (2018). The Structure of Suicidal Beliefs: A Bifactor Analysis of the Suicide Cognitions Scale. *Cognitive Ther. Res.*, *43*, 335–344. https://doi.org/10.1007/s10608-018- 9961-2

Clement, U., & Löwe, B. (1996). Fragebogen zum Körperbild (FKB-20), Testmappe mit Handanweisung, Fragebogen und Auswertungsblättern. *Göttingen: Hogrefe*.

Cloitre, M., Shevlin, M., Brewin, C. R., Bisson, J. I., Roberts, N. P., Maercker, A., Karatzias, T., & Hyland, P. (2018). The International Trauma Questionnaire: development of a self-report measure of ICD-11 PTSD and complex PTSD. *Acta psychiatrica Scandinavica*, *138*(6), 536–546. https://doi.org/10.1111/acps.12956

Cohen, S., Kamarck, T., & Mermelstein, R. (1983). A Global Measure of Perceived Stress. *Journal of Health and Social Behavior*, *24*(4), 385. https://doi.org/10.2307/2136404

Collegium Internationale Psychiatriae Scalarum (CIPS) (2005). Hamilton Depression Scale (HAMD). In G. Weyer, Collegium Internationale Psychiatriae Scalarum (Hrsg.), *Internationale Skalen für Psychiatrie* (5. Aufl., S. 261-268). Göttingen: Beltz-Test.

Crowne, D. P., & Marlowe, D. (1960). A new scale of social desirability independent of psychopathology. *Journal of Consulting Psychology, 24*(4), 349–354. https://doi.org/10.1037/h0047358

Döring, N., & Bortz, J. (1993). Psychometrische Einsamkeitsforschung: Deutsche Neukonstruktion der UCLA Loneliness Scale. *Diagnostica.*

Dybek, I., Bischof, G., Grothues, J., Reinhardt, S., Meyer, C., Hapke, U., John, U., Broocks, A., Hohagen, F., & Rumpf, H. J. (2006). The reliability and validity of the Alcohol Use Disorders Identification Test (AUDIT) in a German general practice population sample. *Journal of studies on alcohol*, *67*(3), 473–481. https://doi.org/10.15288/jsa.2006.67.473

First, M. B., Williams, J. B. W., Benjamin, L. S., & Spitzer, R. L. (2015). *Structured clinical interview for DSM-5 personality disorder*. American Psychiatric Association.

First, M.B., Williams J.B.W., Karg, R.S., Spitzer, R.L. (2016). *Structured Clinical Interview for DSM-5 Disorders, Clinician Version (SCID-5-CV).* American Psychiatric Association.

Fleming, J. S. & Courtney, B. E. (1984). The dimensionality ofself-esteem: II. Hirarchical facet model for revised measure-ment scales. *Journal of Personality and Social Psychology*, *46*, 404–421.

Flückiger, C., Regli, D., Zwahlen, D., Hostettler, S., & Caspar, F. (2010). Der Berner Patienten- und Therapeutenstundenbogen 2000: Ein Instrument zur Erfassung von Therapieprozessen. *Zeitschrift für Klinische Psychologie und Psychotherapie*, *39*(2), 71–79. https://doi.org/10.1026/1616-3443/a000015

Franz, M., Popp, K., Schaefer, R., Sitte, W., Schneider, C., Hardt, J., Decker, O., & Braehler, E. (2008). Alexithymia in the German general population. *Social Psychiatry and Psychiatric Epidemiology*, *43*(1), 54–62. https://doi.org/10.1007/s00127-007-0265-1

Gierk, B., Löwe, B., Murray, A. M., & Kohlmann, S. (2018). Assessment of perceived mental health-related stigma: The Stigma-9 Questionnaire (STIG-9). *Psychiatry Research*, *270*, 822–830. https://doi.org/10.1016/j.psychres.2018.10.026

Gräfe, K., Zipfel, S., Herzog, W., & Löwe, B. (2004). Screening psychischer Störungen mit dem "Gesundheitsfragebogen für Patienten (PHQ-D)". *Diagnostica*, *50*(4), 171–181. https://doi.org/10.1026/0012-1924.50.4.171

Gross, J. J., & John, O. P. (2003). Individual differences in two emotion regulation processes: Implications for affect, relationships, and well-being. *Journal of Personality and Social Psychology*, *85*(2), 348–362. https://doi.org/10.1037/0022-3514.85.2.348

Hamilton, M. A (1960). Rating scale for depression. *J Neurol Neurosurg Psychiatry*, *23*, 56–62.

Hatcher, R. L., & Gillaspy, J. A. (2006). Development and validation of a revised short version of the Working Alliance Inventory. *Psychotherapy Research*, 16, 12–25.

Heinemann, L. A. J., Zimmermann, T., Vermeulen, A., Thiel, C., & Hummel, W. (1999). A new 'aging males' symptoms' rating scale. *The Aging Male*, *2*(2), 105–114. https://doi.org/10.3109/13685539909003173

Herzberg, P. Y. (2003). Faktorstruktur, Gütekriterien und Konstruktvalidität der deutschen Übersetzung des Aggressionsfragebogens von Buss und Perry. *Zeitschrift für Differentielle und Diagnostische Psychologie*, *24*(4), 311–323. https://doi.org/10.1024/0170-1789.24.4.311

Hupfeld, J., & Ruffieux, N. (2011). Validierung einer deutschen Version der Self-Compassion Scale (SCS-D). *Zeitschrift für Klinische Psychologie und Psychotherapie*, *40*(2), 115–123. https://doi.org/10.1026/1616-3443/a000088

Jelenchick, L. A., Eickhoff, J., Christakis, D. A., Brown, R. L., Zhang, C., Benson, M., & Moreno, M. A. (2014). The Problematic and Risky Internet Use Screening Scale (PRIUSS) for adolescents and young adults: Scale development and refinement. *Computers in Human Behavior*, *35*, 171–178. https://doi.org/10.1016/j.chb.2014.01.035

Jeromin, F., Rief, W., & Barke, A. (2016). Validation of the Internet Gaming Disorder Questionnaire in a Sample of Adult German-Speaking Internet Gamers. *Cyberpsychology, Behavior, and Social Networking*, *19*(7), 453–459. https://doi.org/10.1089/cyber.2016.0168

Klein, E. M., Brähler, E., Dreier, M., Reinecke, L., Müller, K. W., Schmutzer, G., Wölfling, K., & Beutel, M. E. (2016). The German version of the Perceived Stress Scale – psychometric characteristics in a representative German community sample. *BMC Psychiatry*, *16*(1), 159. https://doi.org/10.1186/s12888-016-0875-9

Klinitzke, G., Romppel, M., Hauser, W., Brahler, E., & Glaesmer, H. (2012). [The German Version of the Childhood Trauma Questionnaire (CTQ): psychometric characteristics in a representative sample of the general population]. *Psychother.Psychosom.Med.Psychol.*, *62*(2), 47-51

Kocherscheidt, K., Fiedler, P., Kronmüller, K.-T., Backenstraß, M., & Mundt, C. (2002). Zur empirischen Unterscheidung von Scham und Schuld. *Zeitschrift für Differentielle und Diagnostische Psychologie*, *23*(2), 217–224. https://doi.org/10.1024//0170-1789.23.2.217

Komlenac, N., Siller, H., Bliem, H. R. & Hochleitner, M. (2018). Validation of the Internal Structure of a German-Language Version of the Gender Role Conflict Scale – Short Form. *Front. Psychol.* 9:1161. doi: 10.3389/fpsyg.2018.01161

Komlenac, N., & Hochleitner, M. (2021). Internal structure and invariance analyses across gender of the German-language version of the Male Role Norm Inventory-Short Form. *Psychology of Men & Masculinities*, *23*(1), 86–98. https://doi.org/10.1037/men0000356

Kroenke, K., Spitzer, R. L., & Williams, J. B. W. (2001). The PHQ-9: Validity of a brief depression severity measure. *Journal of General Internal Medicine*, *16*(9), 606–613. https://doi.org/10.1046/j.1525-1497.2001.016009606.x

Kühner, C., Bürger, C., Keller, F., & Hautzinger, M. (2007). Reliabilität und Validität des revidierten Beck-Depressionsinventars (BDI-II). *Nervenarzt, 78*, 651–656. https://doi.org/10.1007/s00115-006-2098-7

Leary, M. R., Kelly, K. M., Cottrell, C. A., & Schreindorfer, L. S. (2013). Construct Validity of the Need to Belong Scale: Mapping the Nomological Network. *Journal of Personality Assessment*, 95(6), https://doi.org/10.1080/00223891.2013.819511

Lemmens, J. S., Valkenburg, P. M., & Gentile, D. A. (2015). The Internet Gaming Disorder Scale. *Psychological Assessment*, *27*(2), 567–582. https://doi.org/10.1037/pas0000062

Levant, R. F., Hall, R. J., & Rankin, T. J. (2013). Male Role Norms Inventory–Short Form (MRNI-SF): Development, confirmatory factor analytic investigation of structure, and measurement invariance across gender. *Journal of Counseling Psychology*, *60*(2), 228–238. https://doi.org/10.1037/a0031545

Levant, R. F., McDermott, R., Parent, M. C., Alshabani, N., Mahalik, J. R., & Hammer, J. H. (2020). Development and evaluation of a new short form of the Conformity to Masculine Norms

Inventory (CMNI-30). *Journal of Counseling Psychology*, *67*(5), 622–636. https://doi.org/10.1037/cou0000414

Löwe, B., Decker, O., Muller, S., Brahler, E., Schellberg, D., Herzog, W., & Herzberg, P. Y. (2008). Validation and standardization of the generalized anxiety disorder screener (GAD-7) in the general population. *Med Care*. *46*(3), 266–74. https://doi.org/10.1097/MLR.0b013e318160d093

Malone, G. P., Pillow, D. R., & Osman, A. (2012). The General Belongingness Scale (GBS): Assessing achieved belongingness. *Personality and Individual Differences*, *52, 3,* 311-316. doi:10.1016/j.paid.2011.10.027

Morf, C.C., Schürch, E., Küfner, A., Siegrist, P., Vater, A., Back, M., Mestel, R., & Schröder-Abé, M. (2017). Expanding the nomological net of the Pathological Narcissism Inventory: German validation and extension in a clinical inpatient sample. *Assessment*, *24*, 419-443.

O'Neil, J. M., Helms, B. J., Gable, R. K., David, L., & Wrightsman, L. S. (1986). Gender-role conflict scale: College men's fear of femininity. *Sex Roles*, *14*(5–6). https://doi.org/10.1007/BF00287583

Pincus, A. L., Ansell, E. B., Pimentel, C. A., Cain, N. M., Wright, A. G. C., & Levy, K. N. (2009). Initial construction and validation of the Pathological Narcissism Inventory. Psychological Assessment, 21, 365–379. https:// doi. org/ 10. 1037/ a0016 530

Popp, K., Schäfer, R., Schneider, C., Brähler, E., Decker, O., Hardt, J., & Franz, M. (2008). Faktorstruktur und Reliabilität der Toronto-Alexithymie-Skala (TAS-20) in der deutschen Bevölkerung. *PPmP - Psychotherapie · Psychosomatik · Medizinische Psychologie*, *58*(5), 208–214. https://doi.org/10.1055/s-2007-986196

Raes, F., Pommier, E., Neff, K. D., Van Gucht, D. (2011). Construction and Factorial Validation of a Short Form of the Self-Compassion Scale. *Clin. Psychol. Psychother*. *18*, 250–255. https://doi.org/10.1002/cpp.702

Reidy, D. E., Berke, D. S., Gentile, B., & Zeichner, A. (2014). Man enough? Masculine discrepancy stress and intimate partner violence. *Personality and Individual Differences*, *68*, 160–164.

Reidy, D. E., Brookmeyer, K. A., Gentile, B., Berke, D. S., & Zeichner, A. (2016). Gender role discrepancy stress, high-risk sexual behavior, and sexually transmitted disease. *Archives of sexual behavior*, *45*(2), 459-465.

Rice, S. M., Fallon, B. J., Aucote, H. M., & Möller-Leimkühler, A. M. (2013). Development and preliminary validation of the male depression risk scale: Furthering the assessment of depression in men. *Journal of Affective Disorders*, *151*(3), 950–958. https://doi.org/10.1016/j.jad.2013.08.013

Rosen, R. C., Riley, A., Wagner, G., Osterloh, I. H., Kirkpatrick, J., & Mishra, A. (1997). The international index of erectile function (IIEF): A multidimensional scale for assessment of erectile dysfunction. *Urology*, *49*(6), 822–830. https://doi.org/10.1016/S0090-4295(97)00238-0

Rosenberg, M. (1965). Rosenberg self-esteem scale (RSE). Acceptance and commitment therapy. Measures package, 61(52), 18.

Roth, M. (2003). Die deutsche Version des AISS: Psychometrische Kennwerte und Befunde zur Reliabilität und Validität. *Zeitschrift für Differentielle und Diagnostische Psychologie*, *24*(1), 65–76. https://doi.org/10.1024//0170-1789.24.1.65

Rumpf, H. J., Hapke, U., Meyer, C., John, U. (2002) Screening for alcohol use disorders and at-risk drinking in the general population: psychometric performance of three questionnaires. *Alcohol and Alcohoisml* , *37*(3), 261–268

Russell, D., Peplau, L.A., & Cutrona, C.E. (1980). The revised UCLA Loneliness Scale: Concurrent and discriminant validity evidence. *Journal of Personality and Social Psychology*, *39*, 472-480.

Russell, D. W. (1996). UCLA Loneliness Scale (Version 3): Reliability, Validity, and Factor Structure. *Journal of Personality Assessment*, *66*(1), 20–40. https://doi.org/10.1207/s15327752jpa6601_2

Saunders, J. B., Aasland, O. G., Babor, T. F., De La Fuente, J. R., & Grant, M. (1993). Development of the Alcohol Use Disorders Identification Test (AUDIT): WHO Collaborative Project on Early Detection of Persons with Harmful Alcohol Consumption-II. *Addiction*, *88*(6), 791–804. https://doi.org/10.1111/j.1360-0443.1993.tb02093.x

Scheel, C. N., Bender, C., Tuschen-Caffier, B., & Jacob, G. A. (2013). SHAME–Entwicklung eines Fragebogens zur Erfassung positiver und negativer Aspekte von Scham. *Zeitschrift für Klinische Psychologie und Psychotherapie*, *42*(4), 280–290. https://doi.org/10.1026/1616-3443/a000228

Scheel, C. N., Eisenbarth, H., & Rentzsch, K. (2020). Assessment of Different Dimensions of Shame Proneness: Validation of the SHAME. *Assessment*, *27*(8), 1699–1717. https://doi.org/10.1177/1073191118820130

Schoenleber, Michelle & Roche, Michael & Wetzel, Eunike & Pincus, Aaron & Roberts, Brent. (2015). Super Brief-Pathological Narcissism Inventory (SB-PNI).

Spangenberg, L., Glaesmer, H., Hallensleben, N., Schönfelder, A., Rath, D., Forkmann, T., & Teismann, T. (2019). Psychometric properties of the German version of the suicide cognitions scale in two clinical samples. *Psychiatry Research*, *274*, 254–262. https://doi.org/10.1016/j.psychres.2019.02.047

Spitzer, R. L., Kroenke, K., Williams, J. B. W., & Löwe, B. (2006). A Brief Measure for Assessing Generalized Anxiety Disorder: The GAD-7. *Archives of Internal Medicine*, *166*(10), 1092–1097. https://doi.org/10.1001/archinte.166.10.1092

Stöber, J. (1999). Die Soziale-Erwünschtheits-Skala-17 (SES-17): Entwicklung und erste Befunde zu Reliabilität und Validität. *Diagnostica*, *45*, 173–177. https://doi.org/10.1026//0012-1924.45.4.173

Tangney, J. P., Wagner, P., & Gramzow, R. (1989). The Test of Self-Conscious Affect. *George Mason University, Fairfax, VA*.

Teismann, T., Forkmann, T., Glaesmer, H., Juckel, G., & Cwik, J. C. (2021). Skala Suizidales Erleben und Verhalten (SSEV): Faktorstruktur und psychometrische Eigenschaften. *Diagnostica*, *67*(3), 115–125. https://doi.org/10.1026/0012-1924/a000269

Tylka, T. L., Bergeron, D., & Schwartz, J. P. (2005). Development and psychometric evaluation of the Male Body Attitudes Scale (MBAS). *Body Image, 2*, 161–175.

Tylka, T. L., & Wood-Barcalow, N. L. (2015). The Body Appreciation Scale-2: Item refinement and psychometric evaluation. *Body Image*, *12*, 53–67. https://doi.org/10.1016/j.bodyim.2014.09.006

Vogel, D. L., Wade, N. G., & Haake, S. (2006). Measuring the self-stigma associated with seeking psychological help. *Journal of Counseling Psychology*, *53*(3), 325–337. https://doi.org/10.1037/0022-0167.53.3.325

von Collani, G., & Herzberg, P. Y. (2003). Zur internen Struktur des globalen Selbstwertgefühls nach Rosenberg. Zeitschrift für Differentielle und Diagnostische Psychologie, 24(1), 9-22.

Walther, A., Grub, J., Ehlert, U., Wehrli, S., Rice, S., Seidler, Z. E., & Debelak, R. (2021). Male depression risk, psychological distress, and psychotherapy uptake: Validation of the German version of the male depression risk scale. *Journal of Affective Disorders Reports*, 4. https://doi.org/10.1016/j.jadr.2021.100107

Werner, R. & von Collani, G. (2014). Deutscher Aggressionsfragebogen. *Zusammenstellung sozialwissenschaftlicher Items und Skalen (ZIS).* https://doi.org/ 10.6102/zis52

Wilmers, F., Munder, T., Leonhart, R., Herzog, T., Plassmann, R., Barth, J., & Linster, H. W. (2008). Die deutschsprachige Version des Working Alliance Inventory – short revised (WAI-SR) – Ein schulenübergreifendes, ökonomisches und empirisch validiertes Instrument zur Erfassung der therapeutischen Allianz. *Klin. Diagnostik u. Evaluation, 1*, 343–358.

Wiltink, J., Hauck, E. W., Phädayanon, M., Weidner, W., & Beutel, M. E. (2003). Validation of the German version of the International Index of Erectile Function (IIEF) in patients with erectile dysfunction, Peyronie's disease and controls. *International Journal of Impotence Research*, *15*(3), 192–197. https://doi.org/10.1038/sj.ijir.3900997

Wittchen, H.-U. & Pfister, H. (1997). *Diagnostisches Expertensystem für psychische Störungen (DIA-X).* Frankfurt a.M.: Swets & Zeitlinger.
